# Trait Impulsivity Is Associated with the Risk of Falls in Parkinson’s Disease

**DOI:** 10.1371/journal.pone.0091190

**Published:** 2014-03-07

**Authors:** Katrijn Smulders, Rianne A. Esselink, Roshan Cools, Bastiaan R. Bloem

**Affiliations:** 1 Department of Neurology, Donders Institute for Brain, Cognition and Behaviour, Radboud University Nijmegen Medical Centre, Nijmegen, The Netherlands; 2 Institute for Studies in Sports and Exercise, HAN University of Applied Sciences, Nijmegen, The Netherlands; 3 Department of Psychiatry, Donders Institute for Brain, Cognition and Behaviour, Radboud University Nijmegen Medical Centre, Nijmegen, The Netherlands; 4 Centre for Cognitive Neuroimaging, Donders Institute for Brain, Cognition and Behaviour, Radboud University Nijmegen, Nijmegen, The Netherlands; Hospital General Dr. Manuel Gea González, Mexico

## Abstract

**Objective:**

Impulsivity is a “tendency to act prematurely without foresight.” Clinical experience suggests that such impulsive behavior can impact on the fall risk in Parkinson’s disease (PD), but this has never been tested. We investigated whether trait impulsivity is related to fall risk in a large cohort of PD patients. We also investigated whether trait impulsivity affects the fall risk differently for patients with more or less postural instability and gait disability (PIGD).

**Methods:**

388 patients with PD (H&Y≤3) completed the Barratt Impulsiveness Scale (BIS-11, higher scores indicating greater impulsivity) to assess trait impulsivity, including three subscales: motor impulsivity (e.g. “I do things without thinking”), attentional impulsivity (e.g. “I concentrate easily”) and non-planning (e.g. “I plan tasks carefully”). Falls were registered prospectively for 6 months. Patients classified as non-fallers (0 falls, n = 237) were compared to recurrent PD fallers (>1 fall, n = 78).

**Results:**

Total impulsivity scores were higher for recurrent fallers (59.5) compared to non-fallers (56.8; p = .012). This effect was predominantly driven by higher scores on the subscale for attentional impulsivity (p = .003). The difference in attentional impulsivity was independent of gender, disease severity, dopaminergic medication, and cognitive function. Motor and non-planning impulsivity did not differ between recurrent fallers and non-fallers. There was no evidence that impulsivity modulated the association between PIGD and fall risk.

**Discussion:**

This is the first evidence that impulsivity, in particular in the attentional domain, is related to fall risk in PD.

## Introduction

Falls in Parkinson’s disease (PD) are common and incapacitating [1]. Considering the hallmark motor symptoms of PD, the high fall rate is understandable. However, not all patients with postural instability or gait disability fall, perhaps because these patients compensate by moving more cautiously. In contrast, frequent fallers might miss such adaptive behavior, perhaps due to lack of insight or impulsivity [2]. Indeed, Ahlskog stated that “…some of the worst fallers are those who impulsively jump from their chair or turn without thinking” [3]. Quinn coined the term “motor recklessness” to describe such behavior, which is common in patients with progressive supranuclear palsy [4]. There is as yet, however, no quantitative proof for this clinical observation.

Impulsivity is a complex concept, including “actions that are poorly conceived, prematurely expressed, unduly risky, or inappropriate to the situation and that often result in undesirable outcomes” [5]. Our primary aim was to investigate whether trait impulsivity is associated with fall risk in PD patients. To this end, we assessed trait impulsivity using the Barratt Impulsiveness Scale 11 (BIS-11) to assess the personality construct of impulsivity. The BIS-11 distinguishes motor impulsivity (“acting without thinking”) [6], attentional impulsivity (a lack of “focusing on the task at hand” and “thought insertions and racing thoughts”) [7], and non-planning impulsivity (a lack of “futuring or forethought”) [6], [7]. Fall incidents were prospectively monitored for a period of six months in a large cohort of PD patients. As a second aim, we investigated whether trait impulsivity modulates the association between postural instability and gait disability and fall risk.

## Methods

### Ethics Statement

This study was approved by the regional medical ethics committee (CMO region Arnhem-Nijmegen). Written informed consent was obtained from all participants before the first assessment.

### Participants

The included patients are a subset of the 586 PD patients who participated in the ParkFit study, a multicentre, randomized clinical trial that evaluated the effectiveness of a behavioral program to promote physical activity [8]. Eligibility criteria in the ParkFit study were PD according to the UK Brain Bank criteria [9], Hoehn and Yahr (H&Y) ≤3 [10], age between 40 and 75 years, and a sedentary lifestyle. Exclusion criteria were: unclear diagnosis (no gratifying, sustained response to dopaminergic therapy), Mini-Mental State Examination (MMSE) <24 [11], unable to complete Dutch questionnaires, severe co-morbidity, daily institutionalized care, and deep brain surgery.

After exclusion of participants who had no (n = 124) or incomplete BIS-11 questionnaires (n = 16), or incomplete fall records (n = 58), 388 participants were included. There were no significant differences between included and excluded patients with regard to demographic (age, gender, educational level) and disease characteristics (H&Y stage, MMSE). Because recurrent falls are generally viewed as indicative of pathology, whereas single falls can be regarded as occasional falls with uncertain clinical relevance [12]–[14], we excluded all patients with a single fall over 6 months (n = 73) for the primary analysis (see *Falls)*. This resulted in a sample of 315 patients (66% men, 65±8 years). Mean Unified Parkinson’s Disease Rating Scale-III (UPDRS-III) was 33±10, 76% were in H&Y stage 2 (H&Y 1∶2; H&Y 1.5∶3%; H&Y 2.5∶16%; H&Y 3∶5%), and mean MMSE score was 28±2 (Table 1).

**Table 1:** Demographic and clinical measures for fall groups.

	Non-fallers	Recurrent fallers	*P* value
N	237	78	
Age	65±8	65±8	.715
Gender (% M)	69%	56%	.046
Hoehn & Yahr (%)			
1	1%	3%	
1.5	3%	1%	
2	80%	63%	.001
2.5	14%	20%	
3	2%	13%	
UPDRS-III	32±10	37±11	<.001
PIGD	2.6±1.6	3.5±1.7	<.001
MMSE	28±2	28±2	.097
Falls	0	5±7	<.001
LED total	432±399	634±478	<.001
% using DA agonists	51%	65%	.027
LED-agonists	123±226	164±163	.137
Physical activity level (hours/week)	15.6±10.7	17.3±10.7	.227

P values of independent t-tests and chi-square are presented to compare fall groups.

UPDRS-III: Unified Parkinson’s Disease Rating Scale motor examination; PIGD: Postural Instability and Gait Disability; MMSE: Mini-Mental State Examination; LED: Levodopa Equivalent Dose. DA: dopamine.

Items 27–30 of the UPDRS-III (arising from chair, posture, gait, postural stability) were summed to calculate PIGD scores of the participants. Total levodopa dose equivalent (LED) was calculated, pooling different drugs according to the following formula: regular levodopa dose×1+ slow release levodopa×0.7+ bromocriptine×10+ apomorphine×10+ ropinirole×20+ pergolide×100+ pramipexole×100+ [regular levodopa dose+(slow release levodopa×0.7)] ×0.2 if taking entacapone [15]. LED values for dopamine agonists (LED-agonists) were calculated using the same formula excluding the levodopa factors.

The level of physical activity level was assessed with the LASA physical activity questionnaire (LAPAQ), a validated seven day recall of physical activities [16].

### Cognitive Assessment

All participants completed a cognitive test battery to assess attentional set switching (CANTAB intra-extra dimensional set shift (IDED)), spatial working memory (CANTAB SWM test), and verbal fluency (letter fluency) [17], [18].

### Trait Impulsivity

The Dutch version of the Barratt Impulsiveness Scale 11 is a self-report instrument to assess the personality construct of impulsivity [7], [19]. The questionnaire consists of 30 items that are scored on a four point scale (1–4) and that taps into three sub-traits: motor impulsivity (e.g. *“I do things without thinking”*), attentional impulsivity (e.g. “*I concentrate easily*), and non-planning impulsivity (e.g. “*I plan tasks carefully”*). Total impulsivity is calculated as the sum of all items. Higher scores on the BIS-11 indicate greater impulsivity. Previous studies have shown adequate internal consistency with Cronbach’s α of 0.81 in a study using the Dutch BIS-11 [20]. Cronbach’s α of the total BIS score in the present study was 0.75. Cronbach’s alpha for attentional BIS was 0.67, for non-planning BIS 0.63 and for motor BIS 0.38.

### Falls

Falls were registered monthly using an automated system to monitor falls by telephone (Falls Telephone, ASK Community Systems). The Falls Telephone called participants every month and asked them how many times they had fallen in the previous month. The Falls Telephone is a reliable instrument to monitor falls in PD (sensitivity: 100%, specificity: 78%) [21]. All fall entries were verified by a personal telephone call of trained research assistants to further increase specificity. A fall was defined as “an unexpected event in which the participant comes to rest on the ground, floor, or lower level” [22]. To illustrate, falling back in a chair when trying to stand up from a chair was not characterized as a fall, whereas standing upright in front of a chair, losing balance and falling into a chair, was counted as a fall. Participants were classified as non-faller (0 falls over 6 months), single faller (1 fall over 6 months) and recurrent faller (>1 fall over 6 months). These groups differed significantly with regard to UPDRS-III (*p*<.001), H&Y (*p* = .002) and PIGD (*p*<.001). Compared to the non-fallers, single fallers had significantly higher UPDRS-III (*p* = .032) and PIGD scores (*p* = .041), but did not have different H&Y stages (*p* = .809). Compared to the recurrent fallers, single fallers had lower H&Y (*p* = .002) and PIGD scores (*p* = .0049), but these groups did not differ with regard to UPDRS-III scores (*p* = .137).

### Statistical Analysis

Statistical tests on demographic, clinical, cognitive and impulsivity outcomes were carried out comparing non-fallers with recurrent fallers. Independent samples t-tests were used for continuous variables, and Chi-square tests for categorical variables. Effect size was calculated using Cohen’s d for the difference between non-fallers and recurrent fallers in case of significant differences on impulsivity measures. In an additional analysis, we included the single fallers in the group of non-fallers (non-recurrent fallers, ≤1 falls) and compared impulsivity scores of this group with the group of recurrent fallers (>1 falls).

To account for the possible contribution of gender, disease severity (H&Y and PIGD), and dopaminergic medication (LED total and LED-agonists) on impulsivity or fall risk, we constructed four multivariate logistic regression models (forced entry) with fall group (non-fallers vs. recurrent fallers) as the dependent variable. In model 1, total impulsivity and gender were included as independent factors. In model 2 total impulsivity, H&Y and PIGD scores were included as independent factors. In model 3 total impulsivity, LED total and LED-agonists were the independent factors. Finally, we investigated whether fall risk was predicted by impulsivity independent of cognitive function. In this fourth model we added the cognitive tests that were significantly different between fall groups and MMSE score as independent factors together with total impulsivity. These analyses were repeated replacing total impulsivity with subscales that were significantly different between non-fallers and recurrent fallers.

To assess whether impulsivity modulated the effect of PIGD on fall risk, a logistic regression analysis (forced entry method) was applied with fall group as dependent variable, and the interaction term total impulsivity *x* PIGD, total impulsivity and PIGD as independent variables. The independent factors were centered to facilitate the interpretation of the coefficients. This analysis was repeated with subscales that were significantly different between non-fallers and recurrent fallers instead of total impulsivity. Significance was accepted at p<.05 for all analyses.

## Results

### Demographic and Clinical Differences Between Fall Groups (Table 1)

Seventy-eight (25%) participants reported more than one fall in the period of six months. Non-fallers and recurrent fallers were comparable with regard to age and MMSE scores (all *p*’s>.1). Women were more likely to report recurrent falls (*p* = .046). Compared to non-fallers, recurrent fallers had higher H&Y stages (*p* = .001) and higher UPDRS-III and PIGD scores (*p*’s<.001). Regarding dopaminergic medication, recurrent fallers had higher LED values than non-fallers (*p*’s<.001). Although the percentage of recurrent fallers using dopamine agonists was higher than that of non-fallers (*p* = .027), the groups did not differ in LED-agonists (*p* = .137). Recurrent fallers and non-fallers had comparable levels of physical activity (*p* = .227).

### Impulsivity and Fall Risk

Patients with PD who experienced multiple falls scored 2.7 points higher on the total BIS-11 than non- fallers (*t*_1,313_ = −2.54, *p* = .012, Table 2). Of the subscales, only attentional impulsivity was different between recurrent fallers and non-fallers, with 1.2 higher impulsivity scores for the fallers (*t*_1,313_ = −2.83, *p* = .005). Effect sizes were small to medium; Cohen’s *d* was 0.33 for total impulsivity and 0.37 for attentional impulsivity. Motor impulsivity (*t*_1,313_ = −1.22, *p* = .225) and non-planning (*t*_1,313_ = −1.66, *p* = .098) did not differ between fall groups.

**Table 2:** Self-reported impulsivity scores (BIS-11) for fall groups.

	Non-fallers	Recurrent fallers	T	P value	Cohen’s d
Total impulsivity	56.8±8.3	59.5±8.0	−2.54	.01	0.33
Motor impulsivity	18.1±2.8	18.5±2.6	−1.22	.23	−
Attentional impulsivity	14.5±3.4	15.7±3.7	−2.83	.005	0.37
Non-planning	24.3±4.7	25.3±4.6	−1.66	.10	−

P values are presented for comparisons between fall groups using the independent samples t-test.

Cohen’s d indicates effect size (0.2: small effect; 0.5: medium effect; 0.8: large effect).

In an additional analysis we compared impulsivity scores of non-recurrent fallers (consisting of the non-fallers and single fallers) with those of recurrent fallers. The results of this analysis were similar to the primary analysis: Recurrent fallers had higher total (*t*_1,386_ = −2.33, *p* = .020) and attentional impulsivity scores (*t*_1,386_ = −2.42, *p* = .016) than non-recurrent fallers. The groups did not differ on motor (*t*_1,386_ = −1.28, *p* = .203) and non-planning impulsivity (*t*_1,386_ = −1.57, *p* = .116).

### Controlling Gender, Disease Severity, and Dopaminergic Medication

We constructed multivariate regression models to assess whether impulsivity contributed to recurrent fall risk independently of gender, disease severity, and dopaminergic medication (Table 3 and 4). These analyses showed that total impulsivity was an independent predictor of fall risk when gender and disease severity were controlled, with an odds ratio of 1.04 (95% CI: 1.03–1.08 controlling gender; 95% CI: 1.03–1.07 controlling disease severity). In contrast, total impulsivity was not an independent predictor for fall risk when dopaminergic medication was controlled.

**Table 3:** Output parameters of multivariate logistic regression models assessing the association between total impulsivity and fall risk.

		Controlled variable			Total impulsivity			Model
Controlling:		B (SE)	OR	95% CI	B (SE)	OR	95% CI	R^2^ (Nagelkerke)
1. Gender		NS			0.04 (0.02)	1.04	1.01–1.07	0.05
2. Disease severity	H&Y	NS			0.04 (0.02)	1.04	1.01–1.08	0.13
	PIGD	0.27 (0.10)	1.31	1.08–1.60				
3. Medication^a^	LED total	1.13 (0.38)	3.10	1.45–6.64	NS			0.07
	LED-agonists	NS						
4. Cognitive function	MMSE	NS			0.04 (0.02)	1.04	1.01–1.08	0.05
	Verbal fluency	NS						

Output of logistic regression models controlling for gender, disease severity, dopaminergic medication, and cognitive function. H&Y: Hoehn and Yahr stages. PIGD: Postural instability and gait disability. MMSE: Mini-Mental State Examination. LED: levodopa dose equivalent. ^a^ LED values were divided by 1000 for these analyses.

**Table 4:** Output parameters of multivariate logistic regression models assessing the association between attentional impulsivity and fall risk.

		Controlled variable			Attentional impulsivity			Model
Controlling:		B (SE)	OR	95% CI	B (SE)	OR	95% CI	R^2^ (Nagelkerke)
1. Gender		NS			0.10 (0.04)	1.11	1.03–1.19	0.05
2. Disease severity	H&Y	NS			0.10 (0.04)	1.11	1.03–1.19	0.14
	PIGD	0.27 (0.10)	1.31	1.07–1.59				
3. Medication	LED total	1.12 (0.38)	3.06	1.42–6.57	0.09 (0.04)	1.09	1.00–1.18	0.08
	LED-agonists	NS						
4. Cognitive function	MMSE	NS			0.10 (0.04)	1.11	1.03–1.20	0.06
	Verbal fluency	NS						

Output of logistic regression models controlling for gender, disease severity, dopaminergic medication, and cognitive function. H&Y: Hoehn and Yahr stages. PIGD: Postural instability and gait disability. MMSE: Mini-Mental State Examination. LED: levodopa dose equivalent. ^a^ LED values were divided by 1000 for these analyses.

Attentional impulsivity was a consistent, independent contributor to fall risk in all regression models with odd’s ratios between 1.09–1.11 (95% CI: 1.03–1.19 controlling gender or disease severity; 95% CI: 1.00–1.18 controlling medication). Other significant contributors to fall risk were PIGD (in model with total BIS: OR: 1.31, 95% CI: 1.08–1.60; in model with attentional BIS: OR: 1.31, 95% CI: 1.07–1.59) and LED total (in model with total BIS: OR: 3.10, 95% CI: 1.45–6.64; in model with attentional BIS: OR: 3.06, 95% CI: 1.42–6.57).

### Cognitive Function

There were no significant differences between recurrent and non-fallers on the cognitive tests assessing attentional set shifting and spatial working memory (*p*’s>.08; Table 5). Recurrent fallers scored significantly lower on verbal fluency compared with non-fallers (*p* = .042). However, logistic regression demonstrated that total BIS (OR: 1.04, 95% CI: 1.01–1.08) and attentional BIS (OR: 1.11, 95% CI: 1.03–1.20) remained independent significant predictors for fall risk when controlled for letter fluency performance and MMSE score (Table 3 and 4).

**Table 5:** Cognitive assessment for fall groups.

		*Non-fallers*	*Recurrent fallers*	*P value*
Attentional set switching	IDED Adjusted errors	52±46	61±49	.168
	IDED Stages completed	8±2	7±2	.201
Spatial working memory	SWM Between errors	41±21	46±21	.083
	SWM Within errors	3±4	3±4	.944
Verbal fluency	Score letter fluency	12±4	11±4	.046

Mean (sd) values for performance on cognitive tests assessing attention, working memory and fluency are compared between fall groups.

IDED: Intra- and extradimensional set shift test.

SWM: Spatial working memory.

### Impulsivity, PIGD, and Fall Risk

To assess whether impulsivity has a larger effect on fall risk for patients with more gait and balance problems, a logistic regression model with independent factors total impulsivity *x* PIGD, total impulsivity and PIGD, and fall group as dependent factor was constructed. Total impulsivity *x* PIGD was not an independent predictor of fall group in this model (*p*<.239). Additionally, we tested the interaction between subscale attentional impulsivity and PIGD as a predictor for fall risk in a similar model. This interaction term was also not a significant predictor of fall risk when the main effects were controlled (*p* = .348).

## Discussion

The present data suggest that trait impulsivity is associated with the risk of falls for patients with PD. Patients who sustained multiple falls within 6 months reported higher impulsivity than non-fallers. In particular, fallers scored higher on attentional impulsivity, although the effect size was small to medium. This difference was independent of gender, disease severity, amount of dopaminergic medication use, and cognitive function. We did not find evidence that impulsivity influenced fall risk differently in patients with high or low PIGD scores.

Attentional impulsivity reflects a tendency to be more sensitive to distraction [7], [19]. If a patient cannot adequately devote attention to gait and postural stability, and is susceptible to distraction, then this likely challenges stability. Hence, an alternative account for our findings is that impaired attention underlies differences between fall groups rather than impulsivity. Indeed, difficulty with sustained attention has been associated with fall risk in PD before [23], and in the current study recurrent fallers scored lower on a test of verbal fluency than non-fallers. To rule out the possibility that attentional deficits could explain our findings, we controlled for differences on this cognitive test and found that the association between impulsivity and fall risk was independent of attentional functions. This finding is in line with a previous study of our group showing that attentional demands operationalized in a dual-task paradigm could not explain fall risk in PD [24]. Moreover, in a study of healthy young subjects, the BIS-11 was found to correlate with performance on a neuropsychological test assessing impulsivity, but not with a measure of sustained attention [25]. Hence, our findings suggest that impulsive behavior of the recurrent fallers represents a different construct than attentional deficits.

Based on prior work [26], [27], motor impulsivity was the most likely candidate to correlate with falls. This aspect of impulsivity reflects the inability to control prepotent, impulsive actions [28]. The only other study evaluating impulsivity and fall risk reported that stroke patients with a history of falls performed more poorly on a task assessing motor impulsivity (bilateral scanning task) [26]. The idea that falling in PD might be related to motor impulsivity came from another study demonstrating that PD patients with predominantly postural instability and gait disability tended to make more impulsive errors in a computerized lab tests (Simon task) compared with tremor-dominant patients [27]. The authors suggested that motor impulsivity in combination with PIGD symptoms makes PD patients extra vulnerable for falls. Our results generally concur with this suggestion. However, impulsivity, whether self-reported or measured with computerized tests in the lab, is well known to be a multifactorial phenomenon [29], [30]. Here we extend this prior work by showing that fall risk is particularly associated with self-reported attentional rather than motor impulsivity. Whether this effect of self-reported attentional impulsivity extends to attentional impulsivity as measured with laboratory computer tests, e.g. in terms of premature responding on a 5 choice task, remains to be determined.

We had expected that impulsive behavior would mainly be risky for patients with greater postural instability and gait disability. However, our findings were not consistent with this hypothesis. We observed that total impulsivity increased fall risk for patients with both higher and lower PIGD scores, evidenced by a non-significant contribution of the impulsivity *x* PIGD interaction term to fall risk. To illustrate the impact of impulsivity, patients with high impulsivity scores (total or attentional) were 1.7 times as likely to fall compared with patients with low impulsivity scores (OR for an interquartile range increase). These findings suggest that impulsive tendencies need consideration in the clinic, even in patients who present with minor axial impairments.

We considered the role of dopaminergic medication, because dopamine replacement therapy, and particularly dopamine agonist dosage, is associated with impulse control disorders (ICD) in PD [31]–[33]. Moreover, the fallers in our study were on a higher dose of dopamine, presumably because of their greater disease severity. Theoretically, this could mean that higher disease severity caused falls and, in parallel, called for more dopaminergic medication, thereby increasing impulsivity. To falsify this explanation, we controlled for dosage of dopaminergic medication, dosage of dopamine agonists and disease severity in our analysis, and this did not change the finding that attentional impulsivity was higher in recurrent fallers compared to non-fallers. However, the addition of total LED values resulted in non-significant associations between total impulsivity and fall risk. Hence, the role of dopamine in impulsive behavior and fall risk needs to be further explored.

The patients of our cohort had to have a sedentary lifestyle in order to be eligible for the study and were in the early to moderate stages of PD. This selection limits generalization to the general PD population. Nevertheless, falls were common in this cohort. This stresses the need to improve identification of patients who are at risk for falls, preferably before the first fall. A second limitation is the use of the BIS questionnaire. The BIS-11 has not yet been validated in a cohort of PD patients. Moreover, we found that the motor BIS had low internal consistency. Validation of the total BIS and its subscales in an independent cohort, representative of the general PD population is therefore warranted. Finally, in a recent study it was found that PD patients with ICD’s score higher on attentional BIS, but not on total BIS, than the ICD negative patients [34]. Extending this finding to our study would suggest that our recurrent fallers might be more at risk for ICD’s. In that regard, it would have been interesting to document ICD’s in our cohort as another dimension of impulsivity. However, the absence of information on ICD status in our cohort does not diminish the validity of our interpretations with regard to the relation between trait impulsivity and falls.

The present study provides the first evidence that trait impulsivity is associated with fall risk in PD. However, impulsivity is a complex multifactorial phenomenon [30]. Future research is needed to further explore different aspects of impulsive behavior in relation to fall risk (see [29] for a theoretical framework).
